# Fourth Branchial Anomaly Presenting with a Lateral Neck Mass in a Neonate

**Published:** 2014-07-10

**Authors:** Tae-Kyung Yoo, Soo-Hong Kim, Ha-Shin Kim, Hyun-Young Kim, Kwi-Won Park

**Affiliations:** 1 Department of Pediatric Surgery, Seoul National University Children’s Hospital, Seoul, Korea; 2 Department of Pediatric Surgery, Pusan National University Children’s Hospital, Yangsan, Korea

**Keywords:** Branchial cleft anomalies, Neonate

## Abstract

Branchial cleft anomalies are an important differential diagnosis in congenital neck masses in infants. The third and fourth branchial anomalies are rare branchial cleft anomalies, which are hard to differentiate. We report here an uncommon case of the fourth branchial anomaly that was presented as an asymptomatic neck mass in a neonate.

## INTRODUCTION

Branchial anomalies are an important differential diagnosis of congenital neck masses, including thyroglossal duct cyst or fistula, dermoid cyst, hemangioma, and lymphangioma. Ninety-five percent of branchial anomalies originate from the second arch, and 1% from the first. The third and fourth branchial anomalies are reported rarely [1]. We report a case fourth branchial anomaly presented as a suddenly increasing neck mass. 

## CASE REPORT

A full-term newborn boy weighing 3.6kg presented with a 1.5cm sized mass on his left anterior neck. Ultrasonography showed an anechoic lesion with strong suspicion of cystic lymphangioma. The pediatrician decided masterly inactivity with a possibility of spontaneous regression. After one week, there was a 5cm increase in the size of the mass. The baby was transferred to our hospital for a pediatric surgeon’s opinion. He had no respiratory difficulty or feeding problem. Neck x-ray showed a cystic mass with an internal air-fluid level in the left anterior neck (Fig.1). MRI showed a cystic mass about4 × 4 × 3.7cm in dimension with a suspected fistulous tract at the level of the left pyriform sinus and an infero-medial beak at the level of cervical esophagus (Fig. 2). A branchial cleft cyst with pyriform sinus fistula, a communicating type esophageal duplication cyst and a lymphangioma were included in the differential diagnoses.

**Figure:**
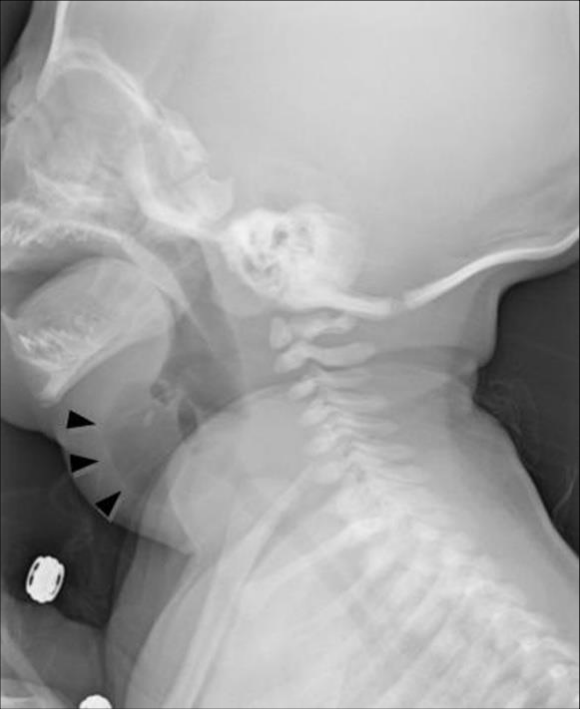
Figure 1: Left lateral neck X-ray finding : An air-fluid level within the mass in the left anterior neck. (Arrows)

**Figure:**
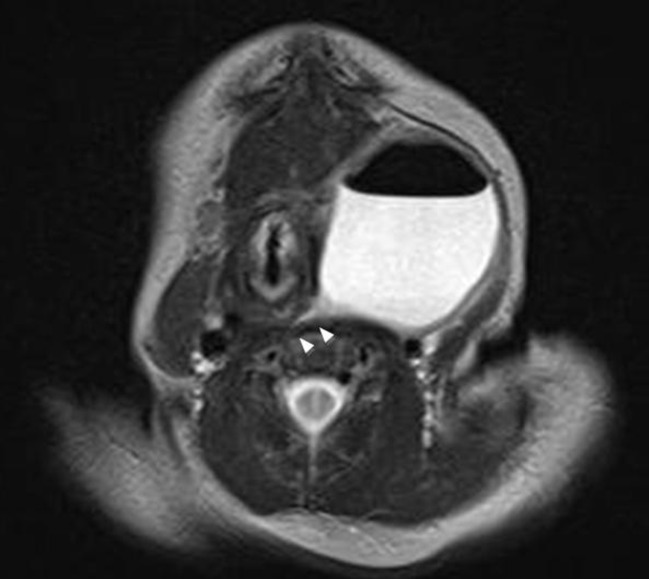
Figure 2: Neck MRI Findings(T2): A 4 × 4 × 3.7 cm size well-defined unilocular cystic mass in the left infrahyoid neck. An internal air-fluid level with suspected fistulous tract at the level of the left pyriform sinus and an inferomedial beak at the level of the cervical esophagus is shown. (Arrows)

At operation, the cystic mass was adhered to adjacent tissues and connected to the pyriform sinus through a 2mm tract. The fistulous tract passed under the superior laryngeal nerve and over the recurrent laryngeal nerve. The mass was excised along with ligation of the fistula tract at its base. Histopathology showed a chronically inflamed benign cyst lined by cuboidal squamous epithelium containing thyroid tissue in the wall and including ectopic thymic tissue (Fig. 3). According to operative and pathological findings, the excised mass was compatible with a fourth branchial cleft cyst. The patient is doing fine on follow-up.

**Figure:**
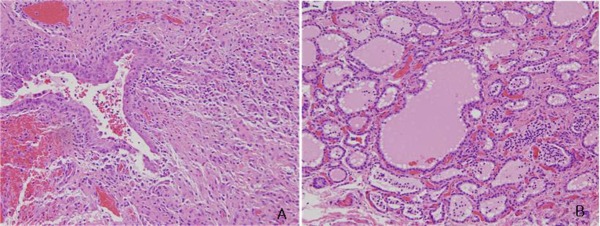
Figure 3: Pathologic findings: A single cuboidal squamous epithelium with partial stratified squamous epithelium and ectopic thymic, thyroid tissue in the wall with chronic inflammation. (H and E, × 100(A), × 400(B))

## DISCUSSION

Embryologically, third branchial arches form thymus gland and lower parathyroid glands, while fourth branchial arches ultimately cover formation of the parafollicular C cells in the thyroid gland and upper parathyroid glands [2,3]. Anatomically, the location of the internal opening into the pyriform fossa is at the cephalad part in the third branchial arches, but at the apex in the fourth branchial arches [1,2]. In addition, a third branchial fistula passes over both the superior and recurrent laryngeal nerves, but a fourth branchial fistula passes under the superior laryngeal nerve and over the recurrent laryngeal nerve [2,4]. Indistinguishing between these two fistulae, the histological difference can be misleading, as accessory thymic tissues derived from the fourth branchial pouch have been described [5]. Also, because of the similarity in their course and post-infectious fibrosis at the time of surgery, precise identification of the internal opening is difficult [2,4,6]. Eventually, diagnosis of the origin of the anomaly is usually made during dissection, where the key difference is the relationship to superior and recurrent laryngeal nerves [4].

In our case, the course of the fistula tract was revealed to pass between the superior and recurrent laryngeal nerves, and the mass contained ectopic thymic tissues and thyroid tissues. The anatomical and histological findings meant that the mass was compatible with a fourth branchial cleft cyst. Although we mentioned a discriminative point of the fourth branchial anomaly compared to a third branchial anomaly, clinically it is not essential to distinguish them, because they have similar symptoms and need the same surgical treatment [4].

The third and fourth branchial anomalies are usually found on the left side of the neck and present variously from nothing to acute suppurative thyroditis, neck abscess and cutaneous discharge. Neonatal cases account for only 8.7% of the fourth branchial anomalies. Over 60% of neonatal cases are presented with respiratory distress[7]. Neonates have been reported to show rapid enlargement of the neck mass as infants swallow, leading to respiratory distress [1,2]. In our case, despite of its asymptomatic presentation, an urgent operation was decided. The treatment of choice for branchial anomalies is complete excision. To avoid recurrence and infection, the tract that makes communication between pharynx and cyst has to be ligated and rarely hemithyroidectomy has to be performed when tract pass through thyroid gland [2,8]. 

In summary, when a lateral neck mass is detected in a neonate, a third/fourth branchial anomaly should be considered in differential diagnosis. As the mass has the risk of a sudden increase in size, which leads to respiratory disturbance or feeding problem, complete excision with tract ligation should be performed in spite of the younger age.

## Footnotes

**Source of Support:** Nil

**Conflict of Interest:** None

